# Evaluation of Antimicrobial Efficacy and Permeability of Various Sealing Materials at the Implant–Abutment Interface—A Pilot In Vitro Study

**DOI:** 10.3390/ma14020385

**Published:** 2021-01-14

**Authors:** Igor Smojver, Marko Vuletić, Dražena Gerbl, Ana Budimir, Mato Sušić, Dragana Gabrić

**Affiliations:** 1 DDM, Private Hospital St. Catherine, 10 000 Zagreb, Croatia; ismojver@gmail.com; 2 DDM, Department of Oral Surgery, School of Dental Medicine, University of Zagreb, 10 000 Zagreb, Croatia; susic@sfzg.hr (M.S.); dgabric@sfzg.hr (D.G.); 3 DM, Clinic of Anesthesiology, University Hospital Clinic Zagreb, 10 000 Zagreb, Croatia; drazena.gerbl@gmail.com; 4 DM, Department of Clinical and Molecular Microbiology, School of Medicine, University of Zagreb, 10 000 Zagreb, Croatia; abudimir@kbc-zagreb.hr

**Keywords:** peri-implantitis, materials, dental implant–abutment interface, prevention

## Abstract

The microenvironment of the oral cavity is altered when an implant, a biocompatible foreign body, is inserted into the mouth. Bacteria settle in the tissues in and around the implant due to the passage of microorganisms through the microgap at the connection of the implant and prosthetic abutment. To prevent colonization of the implant by microorganisms, one idea is to use sealing and antimicrobial materials to decontaminate the implant–abutment interface and close the microgap. The purpose of this study is to evaluate the antimicrobial efficacy and permeability of different types of sealing materials at the implant–abutment interface, under static conditions. Three different sealing material (GapSeal gel, Oxysafe gel and Flow.sil) were used for sealing the implant–abutment interfaces in 60 titanium dental implants, which were first contaminated with a solution containing *Staphylococcus aureus* and *Candida albicans* for 14 days under an aerobic condition. Results showed that a complete seal against bacterial infection was not formed at the implant–abutment interface, while for fungal infections, only GapSeal material helped to prevent microleakage. Findings of this in vitro study reported that application of sealing material before abutment connection may reduce peri-implant bacterial and fungal population compared with the interface without sealing material.

## 1. Introduction

Modern dental implantology is based on the principle of osseointegration and all current implant systems use biocompatible materials based on titanium, zirconium oxide or tantalum. Over the years, many different shapes and surfaces of implants have been produced, which enable better loading of the implant and increase the area of its surface in contact with the alveolar bone [1]. By inserting the implant, we introduce a biocompatible foreign body into the mouth, which changes the microenvironment of the area of the oral cavity into which it is inserted. Changes in this environment lead to the settlement of microorganisms in the peri-implant mucosa, which has less resistance and weaker vascularization than natural teeth. When coupled with reduced oral hygiene, this results in ideal conditions for the development of peri-implant diseases, especially after biofilm formation [2]. The connection between the implant and the prosthetic abutment is the weakest point from a mechanical point of view, and from a biological point of view it is a microgap. Bacterial infection and biomechanical factors, which are associated with implant overload, are the two main factors, which lead to the development of peri-implant diseases [3,4]. Peri-implant diseases themselves are divided into peri-implant mucositis, when inflammation occurs only at the level of the mucosa, and peri-implantitis, where the inflammatory process also affects the bone [3].

For this reason, preparations that fill the microgap and, therefore, participate in the prevention of peri-implant diseases have appeared on the market over the years. A material called GapSeal (Hager and Werken, Duisburg, Germany) was developed at the University of Dusseldorf. The material is based on a highly viscous silicone base and thymol, which allows it to have long-lasting softness and efficient sealing of the implant interstitial space. Given the impossibility of removal by rinsing, and only being removable by mechanical means, it should provide long-term protection against reinfection from inside the implant. Oxysafe (Hager and Werken, Duisburg, Germany) is a material from the same manufacturer that contains active oxygen molecules, which should provide antimicrobial activity, but the sealing effect is questionable. Flow.sil (Bredent GmbH and Co.KG, Senden, Germany) is a product based on poly-dimethyl-siloxane derivatives that provide stability and rigidity, however, it has questionable antimicrobial activity. Chlorhexidine preparations (CHX) are a broad-spectrum bisguanide antiseptic with proven activity in the prevention and treatment of peri-implant mucositis [5]. To the best of our knowledge, there are no recently published studies that investigate the abilities of fungi to penetrate through these new microgap sealants. As a starting point, this research was done in static conditions. This study could provide a clinical benefit by providing evidence for or against the routine use of microgap sealants. If a particular agent shows a beneficial effect, the next step is to conduct the same study under dynamic loading conditions.

The aim of this study is to evaluate, under static conditions, the antimicrobial efficacy and permeability of different chemical materials designed to seal the microgap found at the implant–abutment interface.

## 2. Materials and Methods

In this study, 60 titanium dental implants and 60 original prosthetic abutments were used and divided into two main groups regarding bacteria and fungi. GC Aadva Standard Implants (GCTech.Europe GmbH, Breckerfeld, Germany) of 4.0 mm diameter, with a conical connection to the prosthetic abutment, and a platform-switch were used.

There were 3 test groups formed (with 6 implants in each) for different sealing materials as follows:

1. GapSeal gel (Hager and Werken, Duisburg, Germany);
2. Oxysafe gel (Hager and Werken, Duisburg, Germany);
3. Flow.sil (Bredent GmbH and Co.KG, Senden, Germany).

One positive control group of 6 implants with chlorhexidine gel (Curasept ADS 350 gel, Curaden International AG, Kriens, Switzerland) and one negative control group without sealants (6 implants) were also formed.

### 2.1. Preparation of Dental Implants

**Figure 1:**
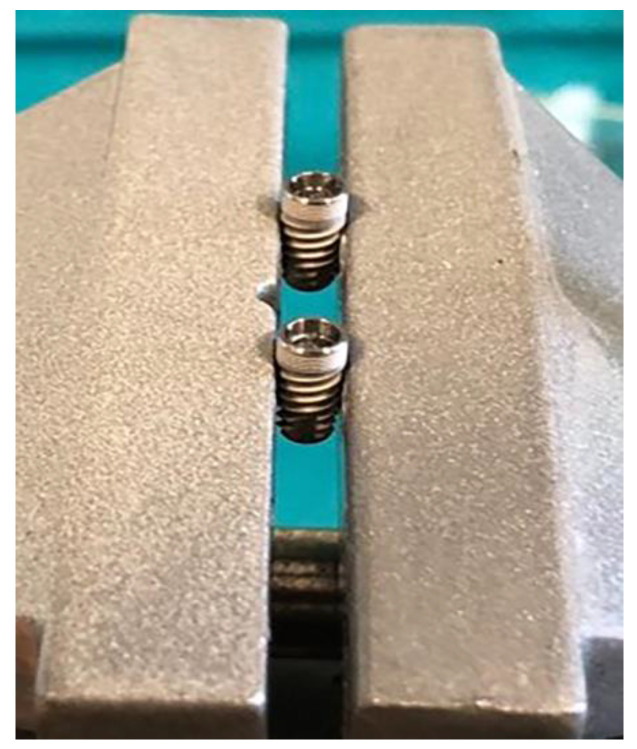
Dental implants placed in a sterile clamp.

**Figure 2:**
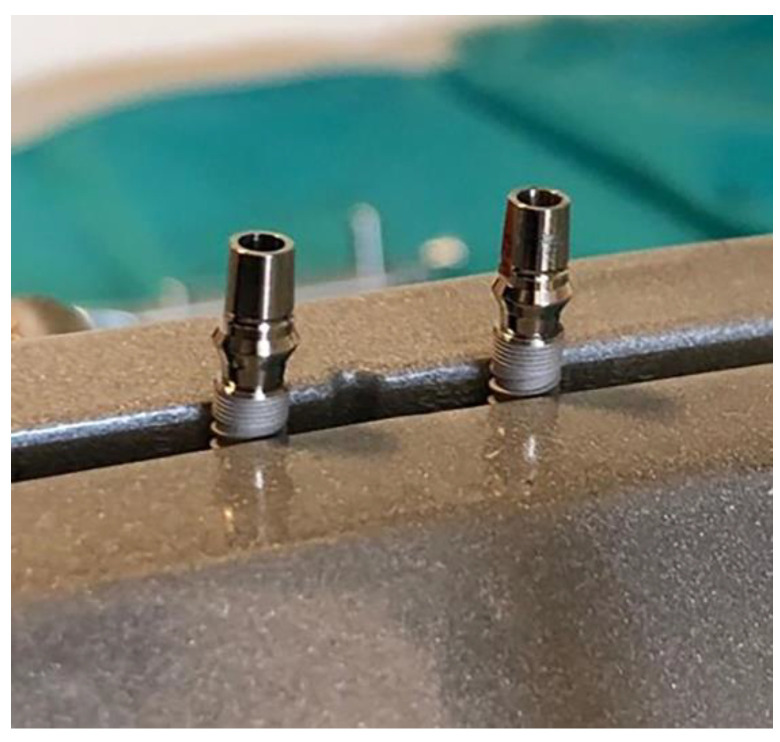
Implant–abutment compound in a sterile clamp.

Dental implants and corresponding original prosthetic abutments were removed from commercial packaging under sterile conditions. After being removed from the sterile package, the dental implants were placed in a vertical position in a sterile clamp using sterile forceps. They were then fixed in a sterile stainless steel clamp (Figure 1) to allow firm swivel action when tightening the prosthetic abutment at 20 N/cm (as recommended by the manufacturer) and to keep the implants in a vertical position (Figure 2).

**Figure 3:**
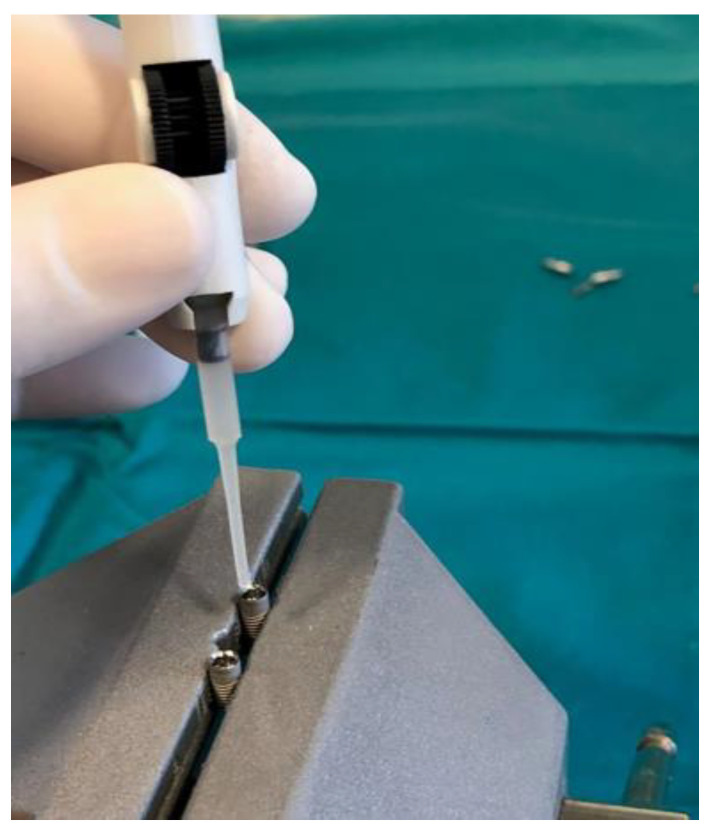
GapSeal gel application.

**Figure 4:**
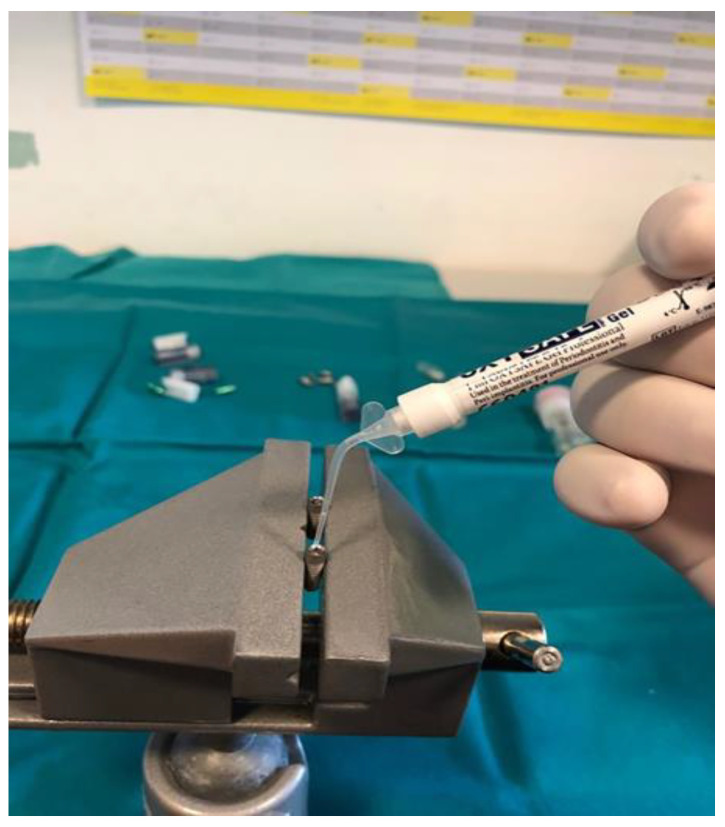
Oxysafe gel application.

**Figure 5:**
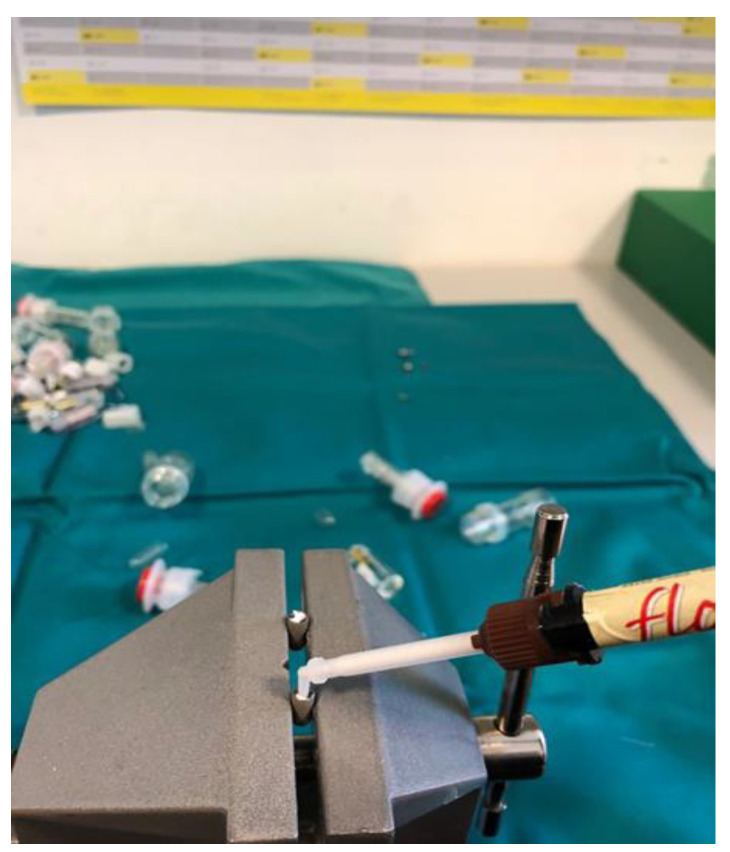
Flow.sil application.

**Figure 6:**
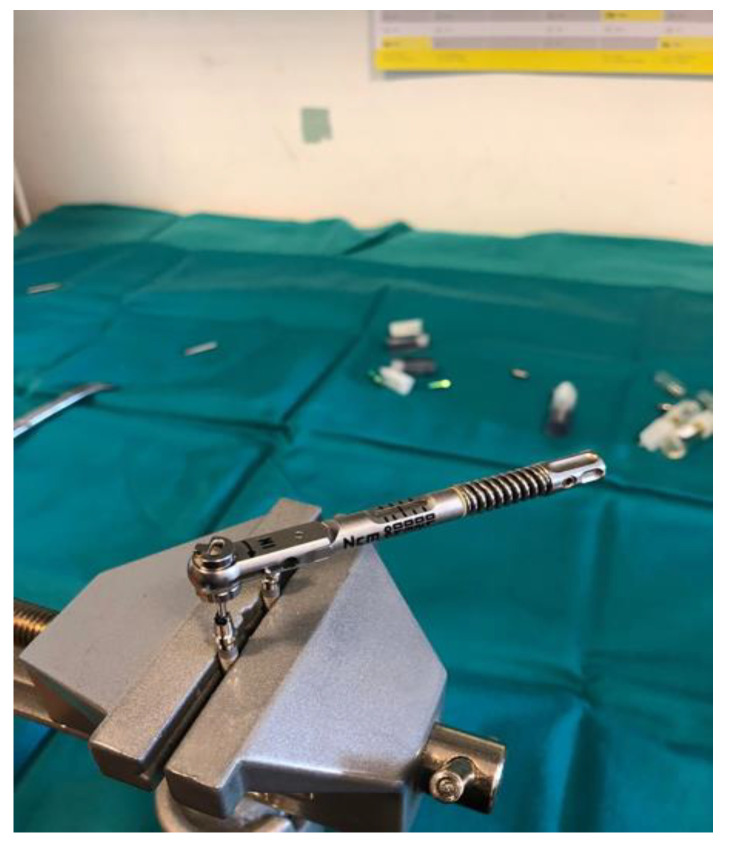
Tightening a prosthetic abutment.

Prior to the installation of the prosthetic abutment, 0.3 µL of sterile brain heart infusion (BHI) solution (brain infusion solids (12.5 g/L), beef heart infusion solids (5.0 g/L), proteose peptone (10.0 g/L), glucose (2.0 g/L), sodium chloride (5.0 g/L) and disodium phosphate (2.5 g/L), pH 7.4 ± 0.2 at 25 °C) was added to the implants, using a sterile micropipette, to serve as a nutrient medium if bacteria and fungi penetrate. The test material was then applied to the edge of the implant, depending on the group (Oxysafe, GapSeal, Flow.sil, CHX gel, Figure 3, Figure 4 and Figure 5), after which a prosthetic abutment was installed according to the manufacturer’s recommendations (20 N/cm for GC Aadva implants (GCTech.Europe GmbH, Breckerfeld, Germany)) (Figure 6). In the case of the negative control group, no test material was applied, but a prosthetic abutment was installed according to the manufacturer’s recommendation.

### 2.2. Contamination of Dental Implants

All microbiological procedures were performed at the laboratory of the Department of Clinical and Molecular Microbiology, University Hospital Centre Zagreb. *Staphylococcus aureus* and *Candida albicans* strains isolated from a clinical sample at the Clinical Hospital Center Zagreb were used for contaminating the dental implants. The bacteria and fungi were grown separately in Columbia Agar for 72 h and then, using thioglycolate broth, a bacterial and fungal suspension was prepared for each of the microorganisms and mixed together in a joint suspension. A density of 600 nm, equivalent of 1 × 10^8^ CFU/mL (colony forming units per milliliter), was set by optical densitometer (Densimat, Biomerieux, Marcy l’Etoile, France).

**Figure 7:**
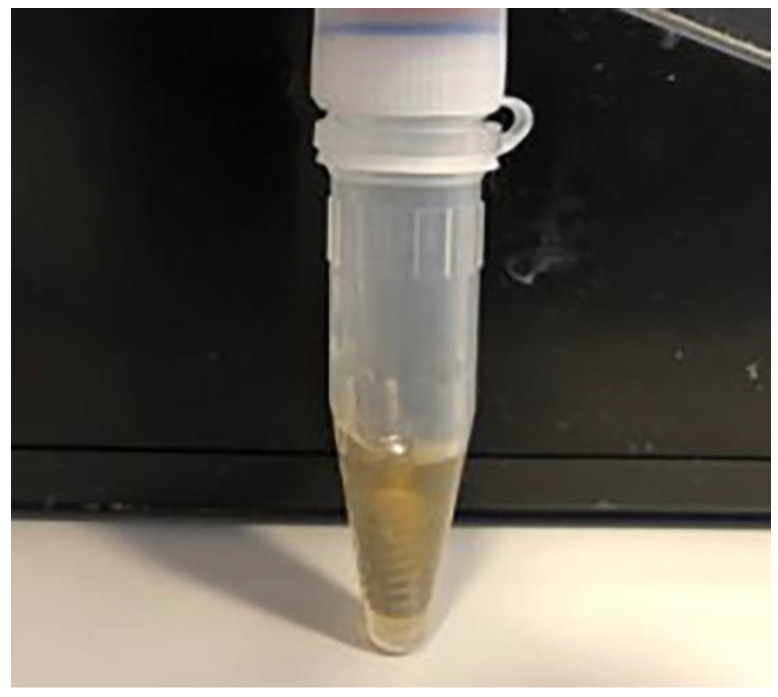
Implant assemblies immersed in a solution contaminated with *S. aureus* and *C. albicans*, in Eppendorf tubes.

All dental implant and prosthetic abutment assemblies were immersed for 14 days, under aerobic conditions, in 300 µL of mixed bacterial and fungal suspension (containing *S. aureus* and *C. albicans* at a density of 0.5 McFarland), which covered the implant neck and abutment (Figure 7).

The opening for screw access remained above the level of the suspension to eliminate the impact of the penetration of the contaminated suspension along the screw itself. No sealant or antiseptic treatment was applied to the negative control group samples, but the implant–abutment assemblies were immersed in the contaminated solution for 14 days, under aerobic conditions with an incubation temperature of 35 °C. The positive control group was treated with an antiseptic gel (CHX gel) and then immersed in the contaminated solution for 14 days, under aerobic conditions.

**Figure 8:**
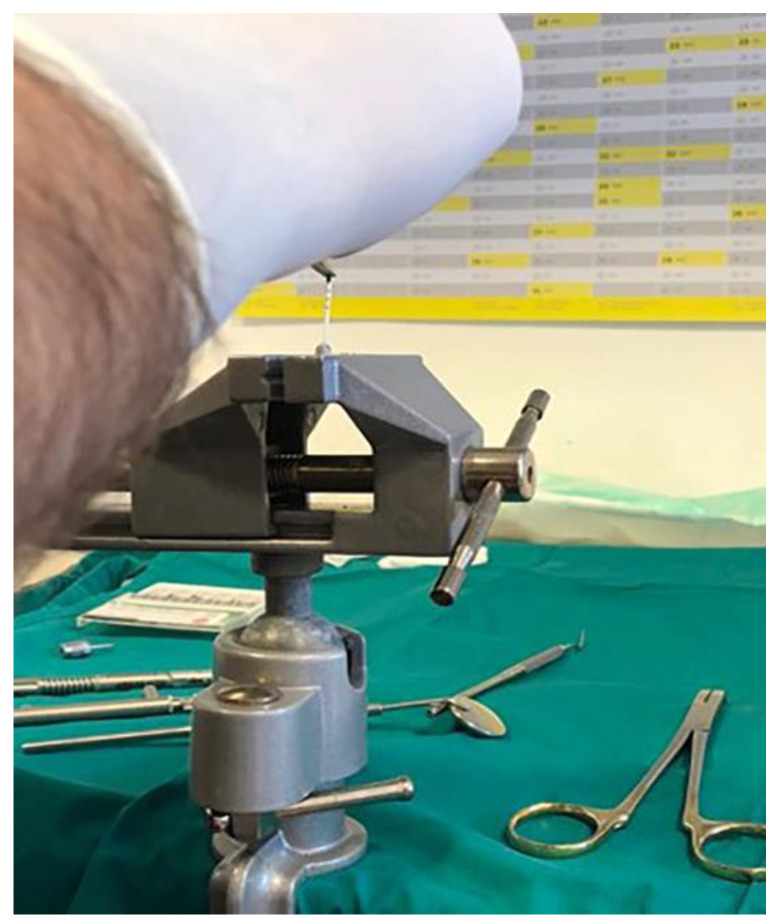
Sampling with paper points.

**Figure 9:**
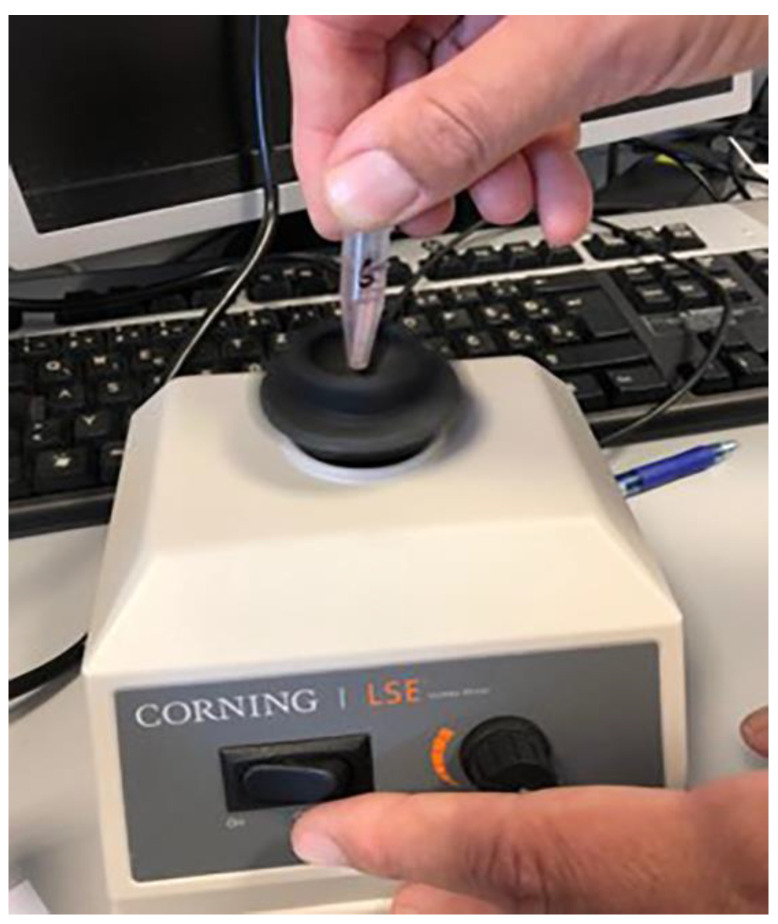
Vortexing.

After 14 days of incubation, the samples were removed from the tubes using sterile forceps, then immersed in 70% alcohol for up to 3 min to prevent external contamination. Samples were then dried with sterile gauze before being carefully disassembled in an upright position, in a sterile clamp. After disassembling the samples, the inner surfaces of the implants were sampled using 3 sterile paper sticks/points (Absorbent points, DENTSPLY Maillefer, Tulsa, OK, USA) (Figure 8), which were then immersed in Eppendorf tubes containing 0.5 mL of sterile phosphate buffered saline (PBS). The contents of the tube, along with the paper sticks, were vortexed for 60 s (Corning^®^ LSE™ vortex mixer, Corning, NY, USA) to remove bacterial and fungal cells (Figure 9).

**Figure 10:**
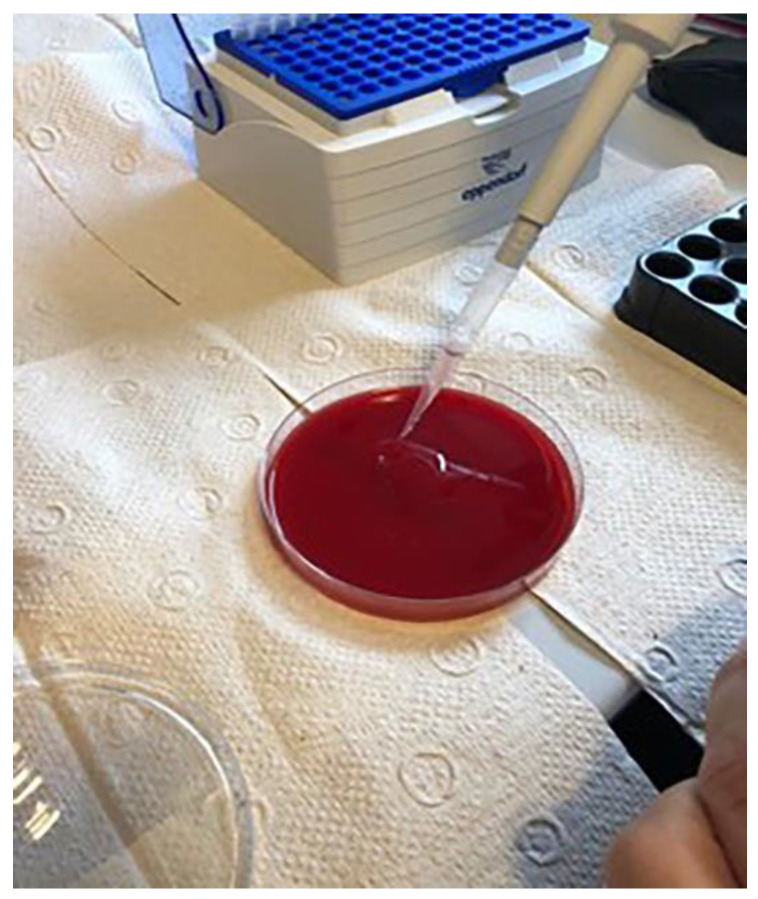
Application of samples on to blood agar.

**Figure 11:**
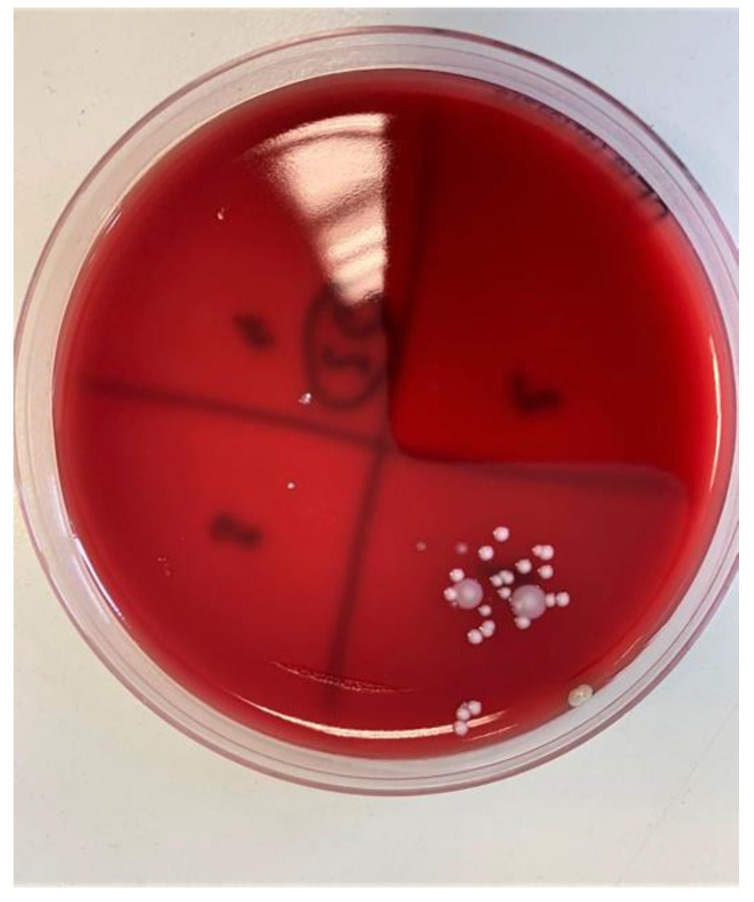
Blood agar plate ready for CFU/mL analysis.

Samples of the complete tube contents were applied to nutrient microbial media with 5% blood agar, then incubated for 48 h at 37 °C (Figure 10). After that, the resulting colonies were identified and quantified (Figure 11). Macroscopically distinctive colonies were confirmed with MALDI Biotyper (Bruker Daltonics, Hamburg, Germany) and the obtained results were entered into the prepared tables.

The results were then determined depending on if there was a presence (positive result) or complete absence (negative result) of bacteria or fungus.

### 2.3. Statistical Analysis

Statistical analysis was performed using the MedCalc software version 19.2.6 (Ostend, Belgium) with the traditional level of statistical significance set at *p* < 0.05.

The efficiency of seals, in comparison to controls, was analyzed using a test for one proportion where proportions of infections with regard to seals were treated as observed, and that with regard to controls as prespecified proportions.

## 3. Results

Using the percentage of infection (Table 1), the seals were compared to controls with regard to *Staphylococcus* spp. (Table 2) and *Candida* spp. (Table 3) infection.

With regard to *Staphylococcus* spp. infection, all seals were significantly more efficient compared to the negative control (*p* < 0.0001). However, only Flow.sil performed as well as the CHX gel treatment used in the positive control group (*p* = 0.01).

With regard to *Candida* spp. infection, the GapSeal was significantly more efficient than both control groups, CHX gel (*p* = 0.02) and negative control (*p* < 0.0001). The third sealant material (Flow.sil) was more efficient only compared to the negative control group (*p* = 0.002). In contrast, the Oxysafe and the Flow.sil were significantly less efficient than the positive control (*p* = 0.0002 and *p* = 0.01, respectively).

## 4. Discussion

This study showed that a complete seal against bacterial infection is not formed at the implant–abutment interface using the tested materials. Regarding fungal infection, only one sealing material helped to prevent microleakage. The presence of other sealing agents helped to reduce microleakage in infections with *Candida* spp. The fungi and bacteria that grew at the implant–abutment interface colonized and percolated through the microgap, then the inner space of implant acted as a reservoir [6]. Analyzing the results presented in this study shows that the concept of a complete hermetic seal at the interface is not possible regarding bacterial infection. These findings are in accordance to several studies [7,8,9]. However, the presence of media at the interface (for example, a gel), reduces the leakage either through having antimicrobial properties or due to a sealing ability. In the negative control group where no sealants were used, the leakage is evidence-based probably due to the lack of complete adaptation between the implant–abutment interface and closing the microgap. Despite this fact, according to Duarte et al. [10], screw tightening is important as time passes as this can influence increased microleakage. Leakage may depend on different methods of tightening the implant–abutment connection and the degree of leakage was found to be dependent on the closing torque. Severity of the leakage has an inverse correlation with the degree of closing torque [11]. In this study, a 20 N/cm torque was used for implant–abutment connection stability, as recommended by the manufacturer for the oral cavity. In cases where the force applied to the implant–abutment interface was bigger than expected, the screw may have loosened, leading to contamination of the inner space of implants.

In 2007, Zitzman and Berglundh [2] concluded that peri-implant mucositis occurs in up to 50% of cases and peri-implantitis in 12–43% of cases. The composition of the biofilm formed on and around the dental implant shifts from that dominated by Gram-positive cocci, to a greater amount of Gram-negative anaerobic and facultatively anaerobic bacteria such as *Aggregaticabacter actinomycetemcomitans*, *Porphyromonas gingivalis*, *Bacteroides forsythus*, *Prevotella intermedia* and *Fusobacterium nucleatum* [2]. Moreover, observational studies have shown that peri-implantitis is more commonly associated with opportunistic pathogens (such as *Pseudomonas aeruginosa* and *Staphylococcus aureus*) [12,13], fungal organisms (such as *Candida albicans*, *Candida boidinii*, *Penicillum* sp., *Rhadotorula sarycesis* and *Pachaces*) [14,15] and viruses (such as human-cytomegalovirus and Epstein–Barr virus) [16] suggesting a rather complex and heterogeneous infection [17,18].

Colonies of the genus *Candida* spp. were found in periodontal pockets, periodontitis and in failed implants in studies by Reynaud et al. [19] and Dahlen et al. [20]. *Candida albicans*, a commensal, is a major pathogen in oral and systemic candidiasis and a major fungus in the oral cavity in 20–40% of healthy individuals [21]. It is considered a major human pathogen in clinical studies, and the incidence of skin and mucosal fungal infections has increased in recent years. In accordance with these findings, and because there is a lack of studies focused on the effectiveness of different types of sealants against leakage of this type of fungus, we decided to contaminate the inner surface of dental implants with *Candida* spp. Several in vitro and in vivo studies [22,23,24,25] have evaluated the ability of different types of bacteria to penetrate an implant along a microgap with a prosthetic abutment, depending on the geometry of the connection itself. Quirynen et al. [22] described that connections with an external six-fold design are more prone to microorganism invasion. Jansen et al. [23] evaluated the microbial leakage of *Escherichia coli* through 13 different combinations of prosthetic augmentation and implant compounds and showed that internal compounds are more resistant to colonization. Steinebrunner et al. [24] evaluated the bacterial colonization of five implant systems with respect to the number of masticatory cycles. Here, they showed that implants with an internal hexagonal connection are more resistant to bacterial leakage under dynamic loading.

Koutouzis et al. [25] reported that implants with an internal Morse taper connection have minimal interface colonization after incubation in bacterial solutions of *Aggregatibacter actinomycetemcomitans* and *Porphyromonas gingivalis*. Presented in vitro study, dental implants with a conical connection to the prosthetic abutment, and a platform-switch were used. It was confirmed that there is microleakage in all bacterial groups and in most fungal groups. We can conclude from these studies that all types of dental implant connections and prosthetic abutments leak bacteria and fungi along the microgap at their connection.

The biofilm made by different types of microorganisms on the external surface of implants is eliminated by immune mechanisms, while internal colonization can persist and produce an unpleasant, malodorous taste and tissue damage and infections of periodontal tissue [26,27]. Trying to prevent such infiltrations in these regions, Duarte et al. [10] recommended the separate use of silicon sealant and chlorhexidine varnish at the cervical part of dental implant, but this was not effective for more than 35 days, demonstrating that they were not able to prevent microleakage. Nayak et al. [11] used GapSeal and concluded that leakage was reduced because of the viscosity of the gel, which flows easily throughout the interface, resulting in a better seal. This is in agreement with our results from this study, and with those obtained by Podhorsky et al. [28], where GapSeal was also used. In our results we showed that it had a similar effect to chlorhexidine gluconate (CHX) in *Staphylococcus aureus* infection and a better result in *Candida albicans* infection. Duarte et al. [10] showed that a combination of chlorhexidine gluconate with tymol varnish, one of the main components of GapSeal, could reduce the number of microorganisms in the oral cavity for a 45–63 day period, leaving 40% of implant–abutment interfaces intact.

## 5. Conclusions

Based on the findings of this in vitro study, we could conclude that application of GapSeal material before abutment connection might cause total prevention for the infection by *Candida albicans* but complete hermetic seal was not achieved regarding bacterial infection with either of tested sealing materials. Further research is needed to test these materials under dynamic loading conditions.

## Figures and Tables

**Table 1:** Percentage of implant–abutment assemblies that became infected in each group (*n* = 6 in each group).

Microbe	Sealant Material			Controls	
	S_1_	S_2_	S_3_	Positive (CHX)	Negative (No Seal)
*Staphylococcus* spp.	66.7	83.3	33.3	66.7	100.0
*Candida* spp.	0.0	83.3	66.7	33.3	91.7

S_1_—GapSeal, S_2_—Oxysafe, S_3_—Flow.sil.

**Table 2:** Comparison between sealant materials and control groups with regard to *Staphylococcus* spp. infection.

Seal	Controls					
	Positive (CHX)			Negative (No Seal)		
	*z*	95% CI	*p*	*z*	95% CI	*p*
S_1_	0.0	34.9; 90.1	1.00	36.4	34.9; 90.1	<0.0001 *
S_2_	1.2	51.6; 97.9	0.22	18.2	51.6; 97.9	<0.0001 *
S_3_	2.5	9.9; 65.1	0.01 *	73.0	9.9; 65.1	<0.0001 *

* Statistically significant; 95% CI—95% confidence interval, *z*-*z* test, S_1_—GapSeal, S_2_—Oxysafe, S_3_—Flow.sil.

**Table 3:** Comparison between sealant materials and control groups with regard to *Candida* spp. infection.

Seal	Controls					
	Positive (CHX)			Negative (No Seal)		
	*z*	95% CI	*p*	*z*	95% CI	*p*
S_1_	2.4	0.0; 26.6	0.02 *	11.5	0.0; 26.6	<0.0001 *
S_2_	3.7	51.6; 97.9	0.0002 *	1.1	51.6; 97.9	0.29
S_3_	2.5	34.9; 90.1	0.01 *	3.1	34.9; 90.1	0.002 *

* Statistically significant; 95% CI—95% confidence interval, *z*-*z* test, S_1_—GapSeal, S_2_—Oxysafe, S_3_—Flow.sil.

## Data Availability

The data presented in this study are available on request from the corresponding author.
